# Evaluating different methods of serum collection to detect failed transfer of passive immunity in newborn calves via refractometry

**DOI:** 10.3168/jdsc.2022-0335

**Published:** 2023-03-02

**Authors:** N. Sonntag, S. Borchardt, W. Heuwieser, R. Sargent, F. Sutter

**Affiliations:** 1 Clinic for Animal Reproduction, Faculty of Veterinary Medicine, Freie Universität Berlin, Königsweg 65, 14163 Berlin, Germany; 2 The Saskatoon Colostrum Co. Ltd. (SCCL), Saskatoon, SK, Canada, S7K 6A2

## Abstract

The objective of the study was to compare 4 different methods of serum collection to assess failed transfer of passive immunity (FTPI) in dairy calves. We hypothesized that centrifuged serum, filtered serum and clotted serum at room temperature, and clotted serum at refrigerator temperature measured with Brix refractometry would highly correlate with IgG concentration assessed by radial immunodiffusion (RID; gold standard) in centrifuged serum. Blood samples were collected from 321 newborn dairy calves. In centrifuged serum (r = 0.88), serum clotted at room temperature (20.2°C ± 6.47; r = 0.86), serum clotted at refrigerator temperature (7.6°C ± 0.91; r = 0.87), and filtered serum (r = 0.70), total solids (TS) in % Brix, and IgG concentrations measured with RID were highly correlated. Regarding the refractometry results among the different serum types, the TS results of serum clotted at room temperature, clotted at refrigerator temperature, and filtered serum showed high correlation coefficients compared with the TS results of centrifuged serum (r = 0.99, r = 0.98, and r = 0.89), respectively. The test characteristics of clotted serum were as accurate as centrifuged serum and generate comparable results. Filtered serum was slightly less accurate. All serum types are valid methods to detect an FTPI in dairy calves, if the specific Brix thresholds for each serum type are considered. Nevertheless, serum clotted at refrigerator temperature should not be the preferred method to avoid the risk of hemolysis.

## Highlights

• Different methods of serum collection can be used to assess FTPI in newborn dairy calves using digital refractometry.
• It is not necessary to centrifugate serum to assess FTPI in newborn dairy calves using refractometry.
• The test characteristics of clotted serum are as accurate as centrifuged serum and generate comparable results, but specific thresholds should be considered for different types of serum.

## Graphical Abstract

**Figure:**
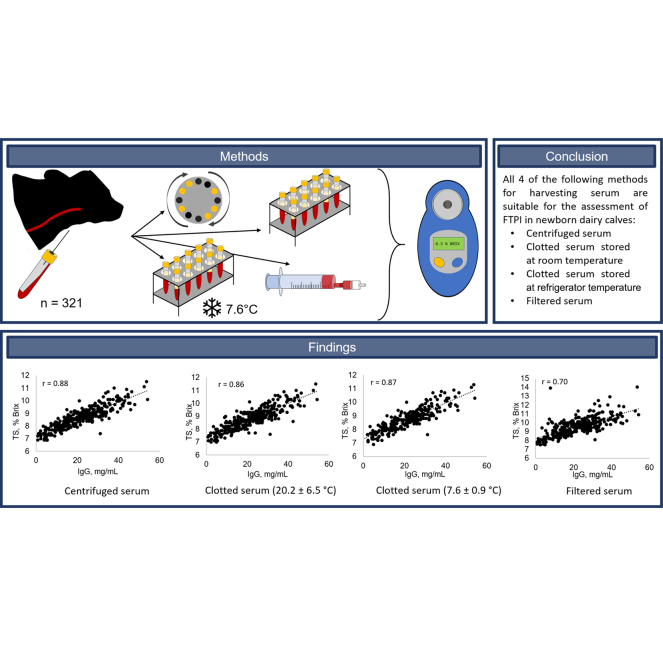
**Summary:** The objective of this study was to evaluate the necessity of centrifuging blood samples and to compare 4 different types of serum collection to detect failed transfer of passive immunity (FTPI) in dairy calves. All different types of serum collection showed high correlations and good test characteristics compared with the gold standard. In conclusion, all 4 methods are suited to evaluate FTPI.

Calves with successful transfer of passive immunity (**TPI**) have greater ability to resist external risk factors and pathogens and have lower preweaning morbidity and mortality (Raboisson et al., 2016; Urie et al., 2018). Furthermore, heifers with a sufficient absorption of colostral IgG have greater ADG and higher milk production in the first 2 lactations (Faber et al., 2005).

The monitoring of TPI to optimize colostrum management is an integral part of heifer rearing. Serum IgG concentrations below 10 mg/mL are classified as failed transfer of passive immunity (**FTPI**) in calves after 24 h of age (Weaver et al., 2000; McGuirk and Collins, 2004; Godden, 2008). Recently, a consensus report (Lombard et al., 2020) recommended a transition from the current dichotomous standard (<10 and ≥10 mg/mL) to 4 categories for serum IgG concentrations (mg/mL): excellent (≥25.0), good (18.0 to 24.9), fair (10.0 to 17.9), and poor (<10.0), which corresponds to FTPI, reaching >40%, ∼30%, ∼20%, and <10% on herd level, respectively.

Several direct or indirect methods of detecting FTPI in newborn calves have been described. The direct assessment of IgG concentration with radial immunodiffusion (**RID**) is still regarded as the gold standard of serum and colostral IgG assessment (Weaver et al., 2000; Wilm et al., 2018). An indirect method estimating IgG measures TS in serum with a digital refractometer. It is a widely accepted analysis method and provides reasonable results if calves are fed with maternal colostrum (Deelen et al., 2014; Elsohaby et al., 2019; Mugnier et al., 2020). Before analyzing the samples, serum must be harvested usually by centrifugation. In practice the collected blood is often left to clot, avoiding centrifugation for reason of practicability. Alternatively, a disposable filter system can be used to evaluate blood samples immediately after collection (da Costa Corrêa Oliveira et al., 2019; Sutter et al., 2020).

The objectives of this study were to compare 4 different types of serum separation (centrifuged, clotted at room and refrigerator temperature, filtered) to assess FTPI in newborn calves. We hypothesized that TS measured in all 4 types of serum were highly correlated with serum IgG concentration assessed by RID in centrifuged serum.

The study was conducted on 2 commercial dairy farms located in Brandenburg, Germany, from May to July 2021 and was in accordance with the Institutional Animal Care and Use Committee of the Freie Universität Berlin (approval number: 2340–15–2021).

The blood samples were collected from clinically healthy male and female Holstein Friesian calves aged 24 h to 7 d. New calves were enrolled weekly into the study. Calves without visible signs of bronchitis, pneumonia, diarrhea, or dehydration were considered clinically healthy. Individuals showing signs of these disorders were excluded from the study.

Through the on-farm documentation, relevant data, such as calf identification, sex, date and time of birth, birth weight, singleton or twin birth, amount of colostrum intake in liters, and colostrum quality in % Brix were acquired and transferred to Excel (Office 2010, Microsoft Deutschland Ltd.). Blood samples were collected into 3 sterile plastic Vacutainer tubes without anticoagulant (1 tube: 9 mL of Vacuette CAT Serum Separator Clot Activator, Greiner Bio-One GmbH; 2 tubes: 3.5 mL of Vacuette CAT Serum Separator Clot Activator, Greiner Bio-One GmbH) via venipuncture of the jugular vein using a 20-gauge, 1.5-inch (3.81 cm) hypodermic needle (Vacuette Multiple Use Drawing Needles, Greiner Bio-One). Four different techniques for blood coagulation and serum separation were used: centrifuged, clotted at room and refrigerator temperature, and filtered.

Serum filtration took place immediately after blood collection by using a disposable filter (2-Drop-Filter, Pharmadoc). Two milliliters of whole blood was aspirated into a disposable syringe (5-mL HS Luer sterile disposable syringe; Henry Schein) and the filter was attached to the cone of the syringe. By applying pressure on the plunger of the syringe, blood was forced through the filter and 1 to 2 droplets of serum were obtained and directly applied to the prism. A new filter was used for each measurement.

The remaining 6 mL of the whole blood samples was centrifuged on the farm at 4,500 × *g* for 10 min at approximately 20°C 1 to 2 h after blood collection to obtain serum. For further analysis, 2 aliquots (1 mL) of centrifuged serum were pipetted into sterile vials (Cryovial 2 mL; Simport) and stored at −18°C. The two 3.5-mL Vacutainer tubes (Vacuette CAT Serum Separator Clot Activator, Greiner Bio-One GmbH) were left to clot via contact activation with the tube surface after blood collection. One tube was stored for 24 h at room temperature (approximately 20°C) and the other tube was stored at refrigerator temperature (approximately 8°C) for 48 h as it took longer for the blood samples to coagulate. With 2 digital thermometers (digital thermometer 30.1017, TFA Dostmann) the ambient temperature (room and refrigerator temperature) was assessed. A time-lapse camera (Brinno TLC 200 Pro, Brinno) was placed in front of both tube racks to record the clotting time. One picture was taken every 10 min.

All of the different serum samples were analyzed with refractometry (% Brix) for the assessment of FTPI. Therefore, 100 µL of centrifuged or clotted serum or 1 to 2 droplets of filtered serum were applied onto the prism of a digital Brix refractometer (Misco PA201, MISCO). The water-soluble solids in serum reflect the impinging LED light. The reflection is converted into a refractive index, which is expressed in % Brix. An integrated thermostat calibrates the refractometer to ensure precise measurements between 0°C and 50°C. The device was calibrated weekly with distilled water at room temperature before the measurement of the samples took place.

The frozen serum aliquots were sent to Saskatoon Colostrum Co. Ltd. in Canada for assessment of the IgG concentration using single RID according to Chelack et al. (1993). In brief, the RID agarose gel plates containing 5 μg/mL anti-bovine IgG with stamped-out wells (2 mm) were used (Bethyl Laboratories Inc.). Serial dilutions of the samples and a reference serum with known IgG concentration were prepared with PBS-Tween buffer and applied into these wells, from where it diffused into the gel. The anti-bovine IgG in the agarose gel reacted with serum IgG resulting in an immunoprecipitin ring. The diameters of these rings were measured using a magnifying loupe and an imaging analyzer. The diameters were compared with a standard curve, which was generated from the reference serum sample with known IgG concentration. Serum samples with lower or higher IgG concentrations than the reference serum were retested with lower or higher dilutions until placement within the range of standards was possible. An internal validation of the method was performed with an intraassay coefficient of variation of 2.1% and an interassay coefficient of variation of 7.2%.

The IgG concentration (mg/mL) determined by RID was plotted against the refractometry results (% Brix) from centrifuged, filtered, and clotted serum (room and refrigerator temperature). Using these distribution plots, Pearson correlation coefficients (r) were calculated. Bland-Altman plots and correlation coefficients were generated with Excel 2010 (Microsoft Deutschland Ltd.). Bland-Altman plots were used to compare the agreement of 2 different measurement methods (centrifuged and clotted serum at room and refrigerator temperature; centrifuged and filtered serum). The difference between the 2 measurements was plotted against the average of these 2 measurements. Limits of agreement were defined as the mean difference ± 1.96 standard deviations (Bland and Altman, 1999; Bland and Altman, 2003). Box plots were created with SPSS for Windows (version 28.0.1.0, IBM Corp.) based on 4 different categories (excellent, good, fair, and poor or FTPI) according to Lombard et al. (2020) and the TS results (% Brix) were compared with the IgG concentrations assessed by RID. Box plots present the 25th percentile as the boundary of the box closest to zero, the mean as line within the box, the median as a cross within the box, and the 75th percentile as the boundary of the box farthest from zero. Minimum and maximum are represented by whiskers. Data falling outside the triple interquartile range are plotted as outliers of the data.

For the 4 serum types, the average % Brix and IgG concentration (means ± SD) and test characteristics [sensitivity (**Se**), specificity (**Sp**), positive predictive value (**PPV**), negative predictive value (**NPV**), and area under the curve (**AUC**)] were calculated using MedCalc software (version 15.6.1, MedCalc). Sensitivity of the test refers to a correct detection of FTPI for a sample with IgG <10 mg/mL. Specificity represents a correct detection of successful TPI for samples with IgG ≥10 mg/mL. The predictive probability of a test result correctly indicating FTPI for a sample with IgG <10 mg/mL is defined as PPV. The predictive probability of a test result correctly indicating a successful TPI for a sample with IgG ≥10 mg/mL is defined as NPV. A receiver operating characteristics curve (**ROC**) was created in which the true positive rate was plotted against the false positive rate in steps of 0.1% Brix. The point on the curve with the maximum value of the Youden's index was defined as the optimal threshold. The overall test characteristic was evaluated based on the AUC according to Swets (1988), who defined AUC = 1 as perfect, 0.9 < AUC < 1 as highly accurate, 0.7 < AUC < 0.9 as very accurate, 0.5 < AUC < 0.7 as accurate, and AUC = 0.5 as noninformative. A statistical difference of the tested variables was considered significant when *P* < 0.05; differences between *P* ≥ 0.05 and *P* ≤ 0.10 were defined as statistical tendencies. The estimated sample size was calculated considering different half-widths (w) and Pearson correlation coefficients (r) based on CIxcorr. CIxcorr is an R-function that defines confidence interval (**CI**) for given Pearson correlation coefficients calculating widths for CI, lower and upper levels, significance α, and sample size n (Moinester and Gottfried, 2014). Assuming a coefficient of correlation of r = 0.80 and w = 0.05, a sample size of 205 samples was required.

Blood was collected from 321 Holstein Friesian calves. All 321 centrifuged serum samples were included in the statistical analyses. A total of 122 (38%) clotted and filtered serum samples were excluded due to hemolysis or failed filtration. Filtration failed when serum was contaminated with red blood cells during the filtering process. In the case of clotted serum, 86 samples (27%) were excluded due to hemolysis, particularly in serum samples stored at refrigerator temperature (n = 83). Thirty-five samples (10.9%) were excluded due to failed filtration. In total 321 centrifuged, 286 filtered serum samples, 318 clotted serum samples at room temperature, and 238 clotted serum samples at refrigerator temperature were analyzed.

Out of 321 calves, 50 samples had IgG concentrations <10.0 mg/mL analyzed with RID, corresponding to a FTPI prevalence of 16%. According to Lombard et al. (2020), the prevalences of the categories excellent, good, and fair were 42% (n = 134), 21% (n = 69), and 21% (n = 68), respectively. The mean IgG concentration was 22.3 mg/mL (±11.12) with a minimum of 0.8 and a maximum of 54.2 mg/mL. The mean for centrifuged serum samples measured with Brix refractometry was 8.7% Brix (±0.84) with a minimum of 6.9 and a maximum of 11.5% Brix. For clotted serum samples the mean was 8.8% Brix (±0.85), ranging from a minimum of 7.0 to a maximum of 11.5% Brix at room temperature and 8.8% Brix (±0.83) with a minimum of 6.9 and a maximum of 11.3% Brix at refrigerator temperature. Filtered serum samples had a mean of 9.6% Brix (±0.97) with a minimum of 7.5 and a maximum of 14.0% Brix.

The mean coagulation time for serum stored at room temperature was 182 min (±3.60) with a minimum coagulation time of 120 min to a maximum of 252 min. The mean room temperature was 20.2°C (±6.47), ranging from a minimum of 10.0°C to a maximum of 27.6°C due to diurnal and nocturnal temperature variations. For samples stored in the refrigerator, blood coagulation took place at a mean coagulation time of 195 min (±2.40) with a minimum coagulation time of 120 min to a maximum of 300 min. The mean refrigerator temperature was 7.6°C (±0.91) with a range from a minimum of 6.4 to a maximum of 9.0°C.

The Pearson correlation coefficient between serum IgG concentrations measured with RID and TS measured with Brix refractometry in centrifuged serum was r = 0.88 (Figure 1a). Similar correlation coefficients were found in serum clotted at room (r = 0.86) and clotted at refrigerator temperature (r = 0.87; Figure 1b and 1c). The correlation of filtered serum with IgG concentrations measured with RID was r = 0.70 (Figure 1d). When using Brix refractometry, the correlation coefficients between TS in centrifuged serum and clotted serum stored at room and refrigerator temperature, and filtered serum were r = 0.99, r = 0.98, and r = 0.89, respectively.

**Figure 1:**
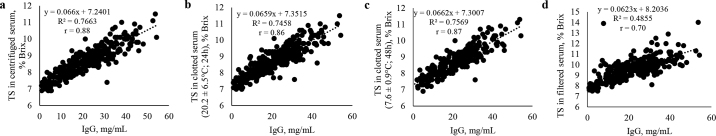
(a) Immunoglobulin G concentration (mg/mL) in centrifuged serum assessed with radial immunodiffusion (RID) compared with TS (% Brix) in centrifuged serum (n = 321; r = 0.88); (b) IgG concentration (mg/mL) in centrifuged serum assessed with RID compared with TS (% Brix) in clotted serum left for 24 h at 20.2 ± 6.5°C (n = 318; r = 0.86); (c) IgG concentration (mg/mL) in centrifuged serum assessed with RID compared with TS (% Brix) in clotted serum left for 48 h at 7.6 ± 0.9°C (n = 238; r = 0.87); (d) IgG concentration (mg/mL) in centrifuged serum assessed with RID compared with TS (% Brix) in filtered serum (n = 286; r = 0.70).

The results of the Bland-Altman plots comparing the difference between TS (% Brix) measured in centrifuged serum and clotted serum (room and refrigerator temperature) and filtered serum were as follows: on average, TS in clotted serum stored at room temperature, at refrigerator temperature, and in filtered serum was 0.10% Brix, 0.05% Brix, and 0.88% Brix higher than in centrifuged serum, respectively. The limits of agreement were −0.35% Brix and 0.13% Brix for clotted serum stored at room temperature, −0.34% Brix and 0.24% Brix for clotted serum stored at refrigerator temperature, and 0.02% Brix and 1.74% Brix for filtered serum, respectively. For the determination of the test characteristics of the different serum types by ROC analysis, the IgG concentration determined by RID was used as the reference (Table 1).

**Table 1:** Test characteristics for TS in % Brix considering centrifuged, clotted, and filtered serum for identification of calves with failed transfer of passive immunity (FTPI; <10 mg/mL in centrifuged serum assessed by radial immunodiffusion as reference value) aged 1 to 7 d

Type of serum	n	Threshold,^1^ % Brix	AUC^2^ (95% CI)	*P*-value	Sensitivity, %	Specificity, %	PPV,^2^ %	NPV,^2^ %
Centrifuged serum	321	8.3	0.96	0.0001	100.0	80.4	49.3	100.0
			(0.93–0.98)					
Clotted serum stored at 20.2 ± 6.5°C for 24 h	318	8.2	0.94	0.0001	89.8	84.8	52.9	97.8
			(0.91–0.97)					
Clotted serum stored at 7.6 ± 0.9°C for 48 h	238	8.3	0.95	0.0001	94.6	80.6	48.1	98.7
			(0.91–0.97)					
Filtered serum	286	9.1	0.86	0.0001	84.8	75.8	40.1	96.3
			(0.81–0.90)					

1 Optimal threshold was determined by receiver operating characteristic (ROC) curve analysis using the threshold with the highest sum of sensitivity and specificity to identify calves with FTPI.

2 AUC = area under the ROC curve; PPV = positive predictive value; NPV = negative predictive value.

Box plots comparing the TPI results in % Brix of different types of serum collection with the IgG concentration measured via RID considering 4 different immunity categories (Lombard et al., 2020) are illustrated in Figure 2. In all 4 types of serum collection, whiskers of the different categories overlapped. For filtered serum, upper quartiles partially overlapped with lower quartiles from the next higher category.

**Figure 2:**
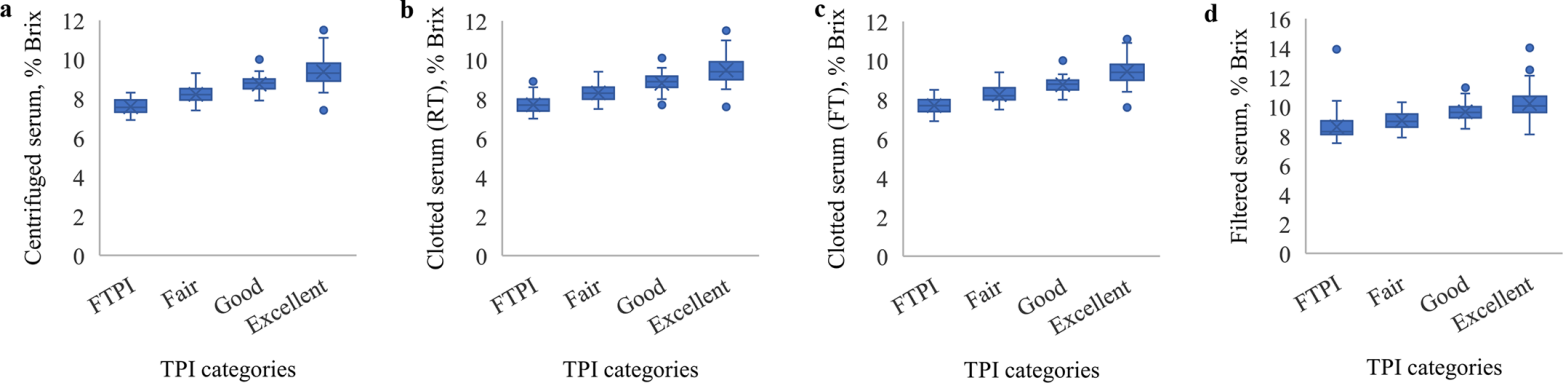
Transfer of passive immunity (TPI) in % Brix within 4 different immunity categories [failed transfer of passive immunity (FTPI) or poor, fair, good, and excellent] defined by Lombard et al. (2020) were illustrated comparing different types of serum collection: (a) centrifuged serum, (b) clotted serum stored at room temperature, (c) clotted serum stored at refrigerator temperature, and (d) filtered serum. In the box plots, the boundary of the box closest to zero indicates the 25th percentile (lower quartile), the cross within the box marks the median, the line within the box marks the mean, and the boundary of the box farthest from zero indicates the 75th percentile (upper quartile). The whiskers represent minimum and maximum without considering extreme values. Data falling outside the triple interquartile range are plotted as outliers of the data.

In centrifuged serum, the correlation (r = 0.88) between serum IgG concentration measured with RID and TS measured with a digital Brix refractometer was consistent with the findings of previous studies (Deelen et al., 2014; Elsohaby et al., 2019; Sutter et al., 2020). Similar correlation coefficients were found in clotted serum stored at room (r = 0.86) and refrigerator temperature (r = 0.87). The correlation coefficients between centrifuged serum and clotted serum stored at room and refrigerator temperature measured with Brix refractometry were r = 0.99 and r = 0.98, which aligns with the findings (r = 0.95) of Wallace et al. (2006). In their study, serum was left to clot and refrigerated afterward. However, the Brix values were not plotted against a gold standard such as RID. Nevertheless, the excellent correlations in both studies support our assumption that both centrifuged serum and noncentrifuged serum are suited for the detection of FTPI by digital Brix refractometry.

The filter system has already been evaluated by da Costa Corrêa Oliveira et al. (2019) and Sutter et al. (2020) with the difference of using heparinized blood rather than whole blood collected in a serum tube. Therefore, the previous studies analyzed plasma instead of serum. In the analysis of filtered serum, the correlation (r = 0.70; 95% CI 0.81–0.90) with IgG concentrations measured with RID was slightly lower than described by Sutter et al. (2020) as r = 0.78 (95% CI 0.86–0.94), suggesting that there are only minimal differences in the different blood media being used. Considering the correlation coefficients between centrifuged serum and filtered serum in % Brix (r = 0.89), our data are consistent with the ones of filtered plasma described by da Costa Corrêa Oliveira et al. (2019), who reported a correlation coefficient of r = 0.92.

The Bland-Altman plots showed a small difference between the 2 storage temperatures of clotted serum samples and centrifuged serum. Clotted serum was 0.11% Brix (room temperature) and 0.05% Brix (refrigerator temperature) higher than centrifuged serum, respectively. In filtered serum, the average difference was slightly higher (0.88% Brix) than in centrifuged serum. The limits of agreement for clotted serum samples were small (−0.35 and 0.13, clotted serum at room temperature; −0.34 and 0.24, clotted serum at refrigerator temperature) due to a small mean difference and standard deviation. The range between the lower and the upper limit of agreement in filtered serum (0.02 and 1.74) was higher than in clotted serum, indicating that the filter is a less reliable, but still acceptable method to detect FTPI. All 4 types of serum were suitable for the detection of FTPI using a digital Brix refractometer. However, it should be kept in mind that filtered serum provides higher % Brix.

As recommended by Swets (1988), we used AUC as an indicator for evaluating the overall test characteristics. For centrifuged or clotted serum, the AUC was >0.9, indicating that these methods were highly accurate to identify calves with FTPI. Centrifuged and clotted serum (room and refrigerator temperature) generated accurate test results (i.e., 95% CI for the AUC overlapped). Clotted and centrifuged serum had high Se and Sp. The test characteristics were in line with the findings of other studies (Morrill et al., 2013; Elsohaby et al., 2015; Mugnier et al., 2020). The optimal thresholds for clotted serum determined by ROC analysis (8.2% Brix at room temperature and 8.3% Brix at refrigerator temperature) were in accordance with the results of other studies (8.3% Brix, Deelen et al., 2014; 8.2% Brix, Elsohaby et al., 2015; 8.4% Brix, Mugnier et al., 2020). In these studies, the optimal thresholds were also determined by using ROC analysis with RID as the reference method.

For filtered serum, the optimal threshold (9.1% Brix) was higher than for centrifuged serum. A similar threshold (9.2% Brix) was determined by using filtered plasma, though using ELISA as the gold standard (da Costa Corrêa Oliveira et al., 2019). Furthermore, the test characteristics for filtered serum showed an AUC of 0.86, and Se and Sp of 84.8% and 75.8%, respectively. These test characteristics were comparable to the findings of Sutter et al. (2020; AUC 0.90, Se 89.8%, and Sp 77.1%) using filtered plasma. Nevertheless, the 95% CI for the AUC of filtered serum did not overlap with the CI of the other serum types, indicating that test accuracy of filtered serum was slightly less accurate than the test accuracy of the other serum types.

In a recent consensus report (Lombard et al., 2020), cutoffs defining 4 categories (i.e., excellent, good, fair, and poor) were suggested for IgG (mg/mL), serum total protein (g/dL), and TS (% Brix). In our study the numbers of calves were classified into the 4 categories based on Brix refractometry and compared with the IgG concentration measured by RID. It is noticeable that the whiskers of the box plots of the different TPI categories overlapped, regardless of the method of harvesting serum. Thus, it is likely that a certain percentage of animals will be misclassified by Brix refractometry. Certainly, on a herd level the implementation of this new standard might help to reduce the risk for mortality and morbidity (Lombard et al., 2020).

As mentioned, 83 samples stored at refrigerator temperature had to be excluded due to hemolysis. Hemolysis in serum samples was also observed by Wallace et al. (2006). However, in their study the prevalence of hemolysis was much lower (6%) and there was no difference between centrifuged and noncentrifuged samples. A reason for serum samples being hemolytic could be a quick cooling before serum samples had started to coagulate. This phenomenon, described as thermal shock by Takahashi and Williams (1983), is damage of red blood cells releasing hemoglobin. Presumably, to avoid hemolysis, the samples should be left to clot 1 to 2 h before cooling down, but further research is warranted.

All 4 serum types (i.e., centrifuged serum, clotted serum at room and refrigerator temperature, and filtered serum) are suitable for the assessment of FTPI in dairy calves. The test characteristics of clotted serum are as accurate as centrifuged serum and generate comparable results. Filtered serum was slightly less accurate to detect FTPI. Nevertheless, centrifuged or clotted serum at room temperature should be preferred over filtered or clotted serum at refrigerator temperature to avoid the risk of hemolysis. Filtered serum can be an acceptable compromise when a centrifuge is not available on farm and the results should be obtained just in time.

## References

[bib1] Bland J.M., Altman D.G. (1999). Measuring agreement in method comparison studies. Stat. Methods Med. Res..

[bib2] Bland J.M., Altman D.G. (2003). Applying the right statistics: Analyses of measurement studies. Ultrasound Obstet. Gynecol..

[bib3] Chelack B.J., Morley P.S., Haines D.M. (1993). Evaluation of methods for dehydration of bovine colostrum for total replacement of normal colostrum in calves. Can. Vet. J..

[bib4] da Costa Corrêa Oliveira L., Borchardt S., Heuwieser W., Rauch E., Erhard M., Sutter F. (2019). Evaluation of a filter system to harvest plasma for identification of failure of passive transfer in newborn calves. J. Dairy Sci..

[bib5] Deelen S.M., Ollivett T.L., Haines D.M., Leslie K.E. (2014). Evaluation of a Brix refractometer to estimate serum immunoglobulin G concentration in neonatal dairy calves. J. Dairy Sci..

[bib6] Elsohaby I., McClure J.T., Keefe G.P. (2015). Evaluation of digital and optical refractometers for assessing failure of transfer of passive immunity in dairy calves. J. Vet. Intern. Med..

[bib7] Elsohaby I., McClure J.T., Waite L.A., Cameron M., Heider L.C., Keefe G.P. (2019). Using serum and plasma samples to assess failure of transfer of passive immunity in dairy calves. J. Dairy Sci..

[bib8] Faber S.N., Faber N.E., McCauley T.C., Ax R.L. (2005). Case study: Effects of colostrum ingestion on lactational performance. Prof. Anim. Sci..

[bib9] Godden S. (2008). Colostrum management for dairy calves. Vet. Clin. North Am. Food Anim. Pract..

[bib10] Lombard J., Urie N., Garry F., Godden S., Quigley J., Earleywine T., McGuirk S., Moore D., Branan M., Chamorro M., Smith G., Shivley C., Catherman D., Haines D., Heinrichs A.J., James R., Maas J., Sterner K. (2020). Consensus recommendations on calf- and herd-level passive immunity in dairy calves in the United States. J. Dairy Sci..

[bib11] McGuirk S.M., Collins M. (2004). Managing the production, storage and delivery of colostrum. Vet. Clin. North Am. Food Anim. Pract..

[bib12] Moinester M., Gottfried R. (2014). Sample size estimation for correlations with pre-specified confidence interval. Quant. Methods Psychol..

[bib13] Morrill K.M., Polo J., Lago A., Campbell J., Quigley J., Tyler H. (2013). Estimate of serum immunoglobulin G concentration using refractometry with or without caprylic acid fractionation. J. Dairy Sci..

[bib14] Mugnier A., Pecceu K., Schelcher F., Corbiere F. (2020). A parallel evaluation of 5 indirect cost-effective methods for assessing failure of passive immunity transfer in neonatal calves. JDS Commun..

[bib15] Raboisson D., Trillat P., Cahuzac C. (2016). Failure of Passive Immune Transfer in Calves: A meta-analysis on the consequences and assessment of the economic impact. PLoS One.

[bib16] Sutter F., Rauch E., Erhard M., Sargent R., Weber C., Heuwieser W., Borchardt S. (2020). Evaluation of different analytical methods to assess failure of passive transfer in neonatal calves. J. Dairy Sci..

[bib17] Swets J.A. (1988). Measuring the accuracy of diagnostic systems. Science.

[bib18] Takahashi T., Williams R.J. (1983). Thermal shock hemolysis in human red cells I. The effects of temperature, time, and osmotic stress. Cryobiology.

[bib19] Urie N.J., Lombard J.E., Shivley C.B., Kopral C.A., Adams A.E., Earleywine T.J., Olson J.D., Garry F.B. (2018). Preweaned heifer management on US dairy operations: Part V. Factors associated with morbidity and mortality in preweaned dairy heifer calves. J. Dairy Sci..

[bib20] Wallace M.M., Jarvie B.D., Perkins N.R., Leslie K.E. (2006). A comparison of serum harvesting methods and type of refractometer for determining total solids to estimate failure of passive transfer in calves. Can. Vet. J..

[bib21] Weaver D.M., Tyler J.W., VanMetre D.C., Hostetler D.E., Barrington G.M. (2000). Passive transfer of colostral immunoglobulins in calves. J. Vet. Intern. Med..

[bib22] Wilm J., Costa J.H.C., Neave H.W., Weary D.M., von Keyserlingk M.A.G. (2018). Technical note: Serum total protein and immunoglobulin G concentrations in neonatal dairy calves over the first 10 days of age. J. Dairy Sci..

